# A virtual simulation experiment platform of subway emergency ventilation system and study on its teaching effect

**DOI:** 10.1038/s41598-022-14968-3

**Published:** 2022-06-24

**Authors:** Sihui Dong, Fei Yu, Kang Wang

**Affiliations:** 1 School of Traffic and Transportation Engineering, Dalian Jiaotong University, Dalian, 116028 China; 2 College of Safety Science and Engineering, Xi’an University of Science and Technology, Xi’an, 710054 China

**Keywords:** Engineering, Civil engineering

## Abstract

For safety engineering majors, it is very important to cultivate the practical ability of professional talents. Due to the difficulty of conducting experiments in dangerous environments, a virtual simulation experiment teaching platform was established. The platform allowed students to understand the structure of the subway ventilation room, and master the control requirements of the ventilation system in the event of sudden fire, blockage, and failure in the subway. Its construction used technologies such as 3D modeling, human–computer interaction, and VR. To test the teaching effect of the simulation experiment platform, two indexes of operating skills and cognitive load were selected to study and analyze the experimental results of students. The research adopts the method of stratified sampling, 46 boys and 10 girls were selected from the first-year students majoring in safety engineering, and they were randomly divided into experimental group and control group, with 23 boys and 5 girls in each group. The experimental group used the simulation platform for teaching, while the control group used the traditional teaching method. The score of the assessment module in the platform was taken as the index of students’ operating skills, and the cognitive load test was carried out by questionnaire to test the teaching effect. The test module scores showed that the average score of the experimental group was 32.79 points higher than that of the control group, and the results of the cognitive load test questionnaire showed that the experimental group scored 35.14% lower than the control group. The research shows that the virtual simulation experiment has a stronger teaching effect than the field experiment.

## Introduction

Because of its punctuality, convenience, and high capacity, the subway is a popular means of transportation for people to move, and it plays an increasingly important role in urban transportation^1^. Due to the special operating environment, relatively closed and complex space, and dense personnel, the subway will cause more serious harm than other transportation modes once fire and other unexpected events occur^2–4^. Therefore, for students majoring in safety engineering, emergency management, and transportation, in the event of a sudden fire and subway blockage in the subway, they need to understand the specific scenarios and ventilation control modes that should be adopted. The traditional teaching method mainly allows students to learn through teachers by imparting professional basic knowledge and displaying relevant pictures. For the scene in the subway in the event of a fire emergency, it is only possible to query the information through imagination and the Internet, it is difficult to understand the internal structure and ventilation equipment and facilities of the subway, and to operate the ventilation control system under different working conditions.

With the development of science and technology, scholars of engineering education put forward the use of virtual reality technology for experimental teaching, in order to enable students to overcome the limitations of the experimental environment, better understand the experiment and master the teaching content. Virtual reality has existed in people’s lives in its current form since the late 1980s. At that time, the virtual reality system was highly complex^5–7^. Later, people combine virtual reality technology with education, and find that this model can greatly improve the attractiveness of learning content and learners’ learning motivation, so virtual reality technology is gradually applied to professional education and training^8–10^. The application of virtual reality technology in teaching is mainly to make participants vividly feel the scenes similar to reality, and bring a more positive impact to participants’ learning. This technology has gradually been studied in training, engineering education, science education, engineering laboratory, etc^11–14^. The virtual simulation platform can provide convenient interactive learning of 3D models by using remote laboratory, computer, and web-based simulation^15,16^.

Virtual experiment teaching platform is gradually becoming an important part of the modern education system. Network virtual experiments can not only improve teachers’ teaching quality, but also improve students’ understanding and motivation levels through virtual simulation and other media combinations^17^. Bourdeau et al. developed simulated agile project management training by using a virtual platform, and the results showed that students’ perceptual learning was positive and the learning experience was stimulating and challenging^18^. Xu et al. used the developed virtual platform for teaching and found that it can improve the quality of teaching and promote the effective learning of students^19^. Chen et al. established a psychological virtual simulation experiment teaching system according to the user needs of different personnel and found that the system has strong practicability^20^. Tiffany et al. studied virtual simulation cases, and the results showed that students improved their understanding ability and interaction ability^21^. He et al. analyzed the development of the virtual simulation experiment teaching platform, and expanded the practical content and training methods of the Unmanned Aerial Vehicle experiment^22^. Jiang et al. took Pingtan Island as a case study to establish a virtual simulation experiment Web3D platform, simulating real field practice scenes to the maximum extent, greatly improving the effect of experimental teaching^23^. Sun et al. studied the differences in learning performance between learners in different environments by comparing the traditional learning environment with the learning environment combined with virtual reality, and found that the learning performance of learners in the virtual reality environment is better^24^. The University of Morocco has evaluated teachers and learners in a virtual learning environment, and the results show that the use of virtual laboratories has a positive impact on learning outcomes^25^. Badilla et al. assessed how teaching and learning processes can be facilitated in a 3D virtual environment^26^. The learning experience in the virtual laboratory is an important way for students to acquire knowledge and practical ability^27^. Therefore, it is an important project to build a virtual laboratory that has a positive impact on students’ learning effect.

At present, there were more and more research on virtual simulation experiment platforms, and there are many types of research on the general risk control system of subway emergencies, but there are few types of research on subway emergencies based on virtual simulation experiment platforms. Chen et al. studied fire escape experiments with virtual reality technology, which provided the reference for risk management and subway emergency evacuation^28^. Hu et al. conducted a simulation study on the most effective cooperative operation mode of the exhaust system in the tunnel track area and the platform ventilation system^29^. Xu et al. developed a fire training simulator that can provide safe paths and applied it to subway stations. The results show that the simulator has a high level of accuracy and smooth interaction functions^30^. However, the existing research on the virtual simulation experiment of ventilation system control under subway emergencies is still very limited. Most of them only study part of the content or are only based on the theoretical level. The experimenter can’t conduct the experiment interactively in the realistic virtual environment, resulting in limited experimental conditions. Virtual reality systems have shown a growing trend in modern science. This modern trend in education focuses on students’ observation ability, creative thinking, and motivation to learn knowledge, these learning effects are usually reflected in the cognitive load and operational skills of students in the learning process. It is generally believed that operating skills reflect students’ practical ability to apply knowledge, while cognitive load reflects students’ understanding and mastery of knowledge. According to cognitive load theory, there are three types of cognitive load, internal cognitive load, external cognitive load, and related cognitive load. The instructional designer can’t have a direct influence on the internal cognitive load, but the external cognitive load and related cognitive load are related to the instructional designer, and the three types of load are superimposed on each other^31,32^. Yang et al. developed a virtual simulation teaching platform for urban rail transit and studied the influence of simulation teaching on students’ knowledge learning and cognitive load^11^. Researchers have found that using a virtual simulation platform to improve teaching design can help reduce students’ cognitive load and promote effective learning^11,17,33^. The traditional teaching method is difficult to ensure the participation of each student and the teaching quality of teachers, and the limitation of emergency experiment has a certain impact on students’ deep learning and understanding, so virtual simulation experiment is adopted to improve students’ learning effect and course satisfaction. Previous studies have also shown that virtual simulation experiment teaching has a positive impact on teacher teaching and student learning^34,35^. Therefore, it is of great significance to examine the effect of a virtual simulation platform.

In view of this, based on the principle of combining and complementing the virtual and the real, this platform uses 3D modeling, VR, and human–computer interaction technologies to construct virtual subway ventilation system facilities and emergency scenes in the context of subway emergencies. The virtual simulation technology is applied to the teaching of subway emergency ventilation system control experiment, and the students’ operating skills and cognitive load are tested through the experimental examination module and questionnaire survey. The purpose is to improve students’ innovation ability, practical operation ability, and learning efficiency of experimental courses, to make up for the lack of three-dimensional structure cognition and human–computer interaction in traditional teaching, to lay the foundation for students to adapt to the work of metro safety production management, emergency command and development of emergency plans, and to promote the training process of high-level applied engineering talents.

## Experimental platform construction

### Experimental platform construction objectives

The main objective of the experimental platform is that students can be familiar with the subway ventilation system, understand the ventilation facilities and equipment of the subway station, ventilation control room and tunnel, and be familiar with the working conditions and possible scenarios under various emergencies that may exist during the operation of the subway. In the aspect of students’ learning practical operation skills, mainly involves the operation of facilities and equipment in the integrated control ventilation system, so as to realize the reasonable organization of smoke flow and ensure the emergency escape of passengers.

The application of the simulation platform, cultivates students’ learning ability and scientific and rigorous learning attitude, and establishes students’ social responsibility for safety, disaster reduction, and emergency management. It will help students quickly adapt to work related to subway safety production management, emergency command, emergency plan formulation, and subway ventilation system design after graduation, and help students’ learning and growth.

### Overview of experimental platform

This virtual simulation experimental teaching platform is based on 3D simulation technology, HTML5 technology, and WebGL technology, using Unity3D, 3D Studio Max, Maya, Photoshop, and other development tools. Using Windows Server operating system and Mysql database, experimental exploration of knowledge points related to the subway ventilation control system mode is realized in an all-around way, and the equipment and facilities of the complex subway ventilation system are intuitively presented in the form of graphics and application scenarios. The experiment does not need to download the program, which ensures the real-time storage requirements of operational data.

### Function of the experimental platform

**Figure 1:**
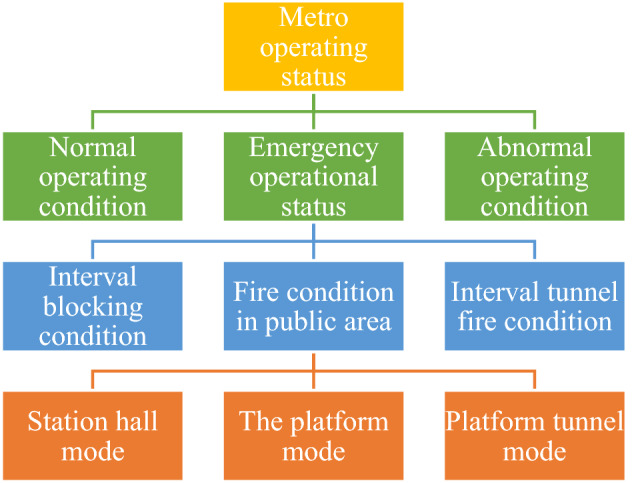
Metro operation status.

The virtual simulation experimental environment constructed by the experimental system is based on the real environment of the Dalian subway station, and the working conditions and models in the system are consistent with the emergency plan set on site. The experiment mainly simulates three operation states in the emergency operation state. Metro operation status mainly includes three categories as shown in Fig. 1, the experiment mainly simulates the ventilation system control under three working conditions in emergency operation.

Before the ventilation system control of subway emergency operation, students need to know the facilities and equipment, including jet fan, station exhaust fan, piston air duct and air valve, detour air duct and air valve, rail head air duct and air valve, rail bottom air duct and air valve, and contact channel, etc. The students carried out the experiment through 13 interactive operation steps, which mainly included equipment cognition interaction, system roaming interaction, and emergency experiment interaction. In the process of interactive operation, students can independently recognize and control the ventilation system equipment many times and can rotate and zoom the equipment 360 degrees. Through understanding and learning, students need to control the combined air valve and fan and other equipment and facilities in the simulation experiment, so as to realize the control of each ventilation mode during emergencies. It will not be affected by the limitations of realistic conditions and can improve the efficiency of students’ learning and teachers’ teaching. In the study of the experimental platform, students can combine the theoretical knowledge of the ventilation system with the practical operation of the system, and learn the relevant knowledge of each facility and equipment while comprehensively viewing the facilities and equipment. In the process of roaming the system, students can learn the internal ventilation structure and evacuation path of the subway by clicking the mouse. During the emergency experiment, students can choose different working conditions for experimental simulation, operate each piece of equipment and watch the overall route of the ventilation system in the simulation process.

Through the simulation study of ventilation system control in case of subway emergencies, students can be trained in their thinking and operation ability of subway ventilation system, cultivate their knowledge application ability, comprehensive practice ability, thinking judgment ability and analysis ability, enhance students’ knowledge learning in subway safety management and subway ventilation system, and lay an important foundation for students’ growth.

## The operation method of the virtual simulation platform

By developing this experimental system, students can understand the subway ventilation control system, the main equipment in the system, and the structure of the ventilation room, master the requirements of the ventilation control system, and establish the relationship between physical facilities and simulation models. This paper introduces the subway ventilation control modes under three working conditions and 13 scenarios designed by the virtual simulation experiment system. The experiment mainly includes five steps: entering the system, experimental preview, equipment cognition, model simulation, and experimental examination.

### Enter system module

**Figure 2:**
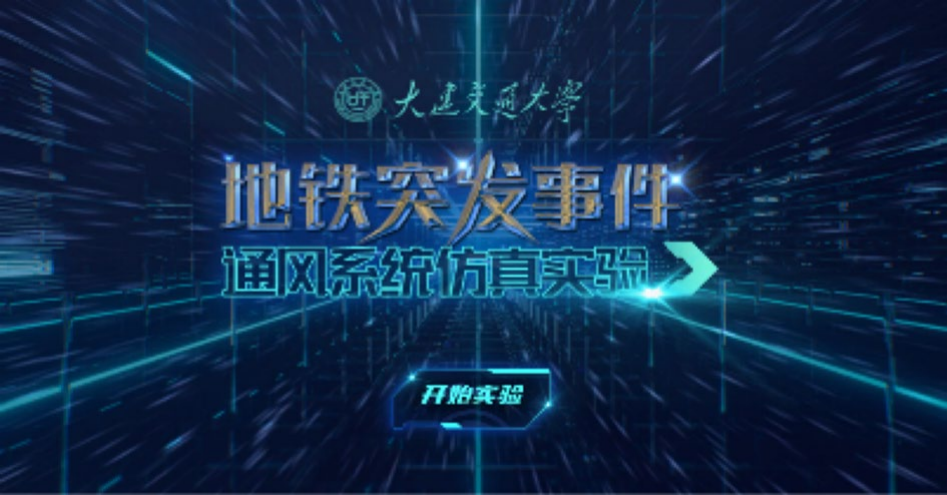
System main page.

**Figure 3:**
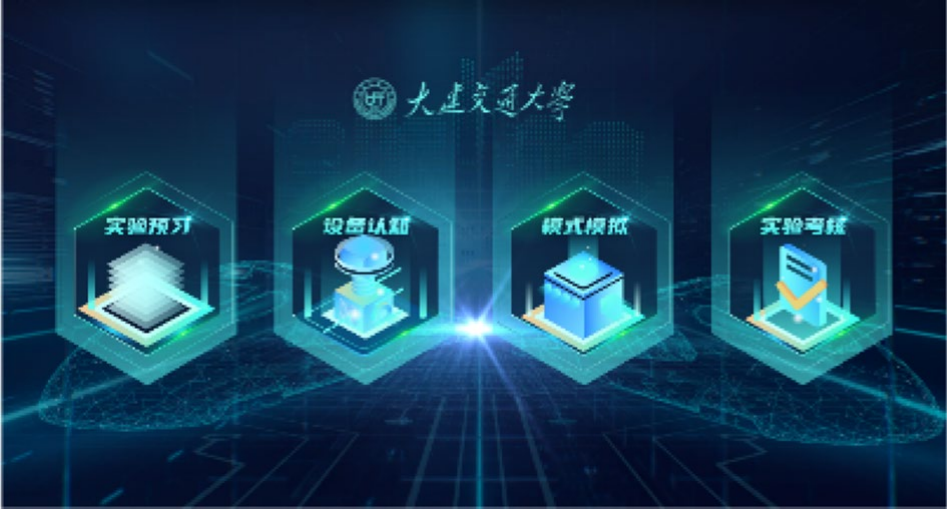
Functional interface of the system.

After entering the experimental system, the main page appears, as shown in Fig. 2.Then click start experiment to display the function interface of the system, as shown in Fig. 3.

The functional interface consists of four modules, experimental preview, equipment cognition, model simulation, and experimental assessment. Selecting the module will enter the corresponding interface. The experiment requires completing the experiment preview first, then completing the equipment cognition, then entering the model simulation, and finally conducting the experimental assessment. After completing one module, you can enter the next module.

### Platform preview module

**Figure 4:**
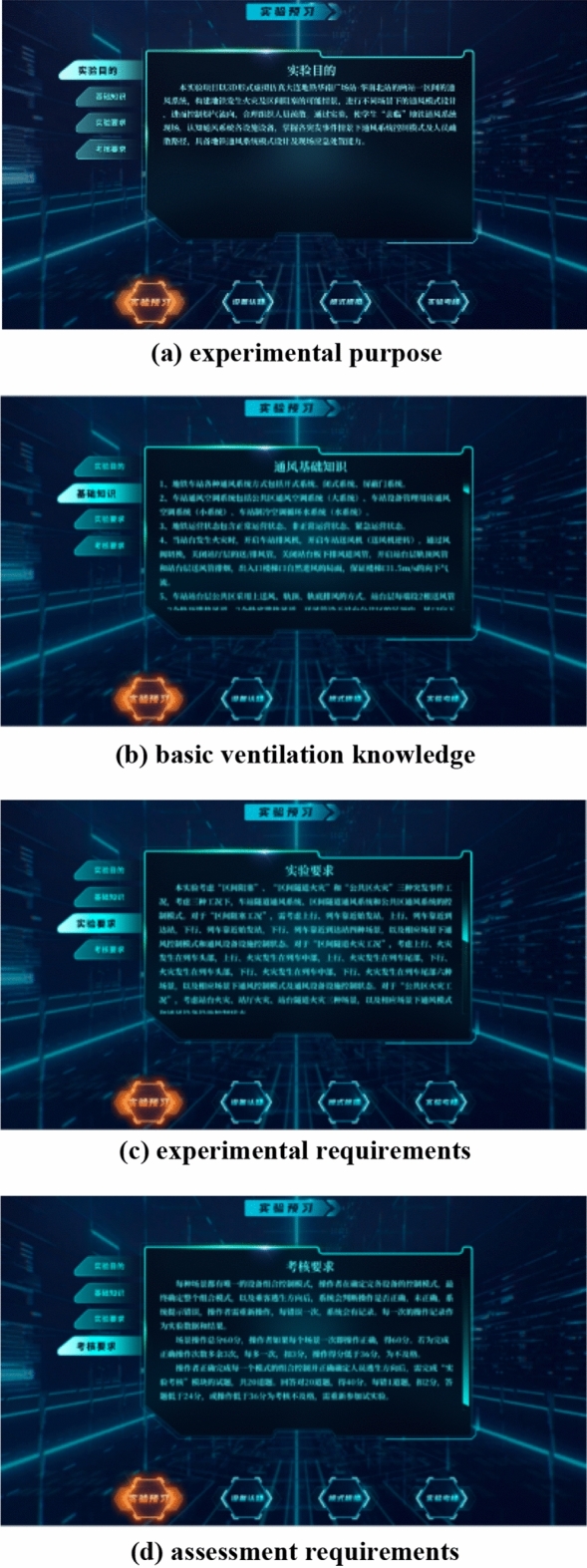
System experiment preview module.

After entering the experimental preview module, the contents to be previewed will appear, including experimental purpose, basic ventilation knowledge, experimental requirements, and assessment requirements, as shown in Fig. 4. The ventilation control mode in the preview module involves 13 scenarios under three working conditions. In different subway scenes, different environments, and sections, the flow speed, and direction of the flue gas were different, and the escape speed of people at different locations were also different. Whether people can escape safely before the danger arrives can be displayed in the system.

### Device cognition module

**Figure 5:**
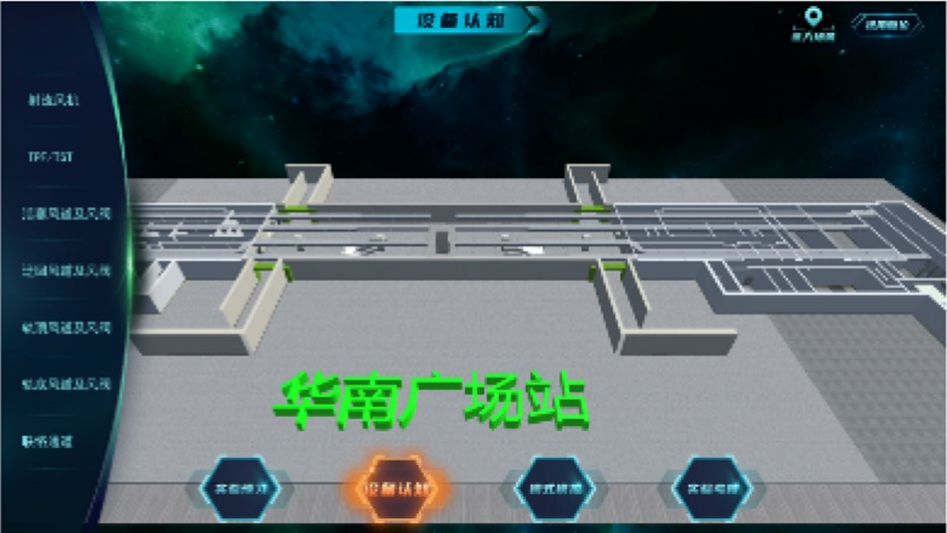
System equipment cognition module.

After completing the experimental preview module, you can enter the equipment cognition module. The facilities and equipment to be recognized include jet fan, TPF, TSF, piston air duct and air valve, detour air duct and air valve, rail top air duct and air valve, rail bottom air duct and air valve, liaison channel, etc., as shown in Fig. 5.

**Figure 6:**
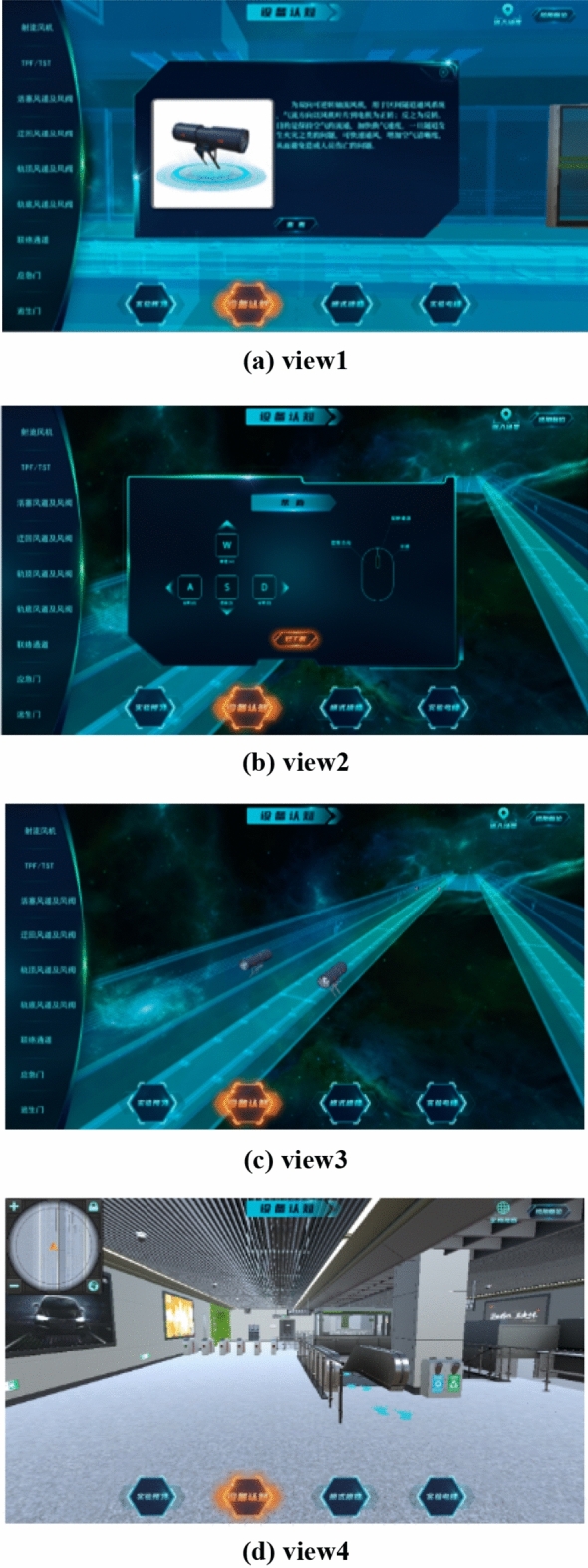
Cognitive process of jet fan.

Students click the device name on the left to display the corresponding basic device introduction and device picture. After clicking view in the device picture, they will prompt the operation mode of viewing during roaming. After understanding, they can see the 3D appearance of the device and the position of the device in the system. Click the entry scene in the upper right corner of the page, and students can roam freely in the scene to view the main facilities and equipment in the system, as shown in Fig. 6d. Click View Angle reset to exit the scene and enter the panoramic view. The cognitive process is taking a jet fan as an example is shown in Fig. 6.

### Mode simulation module

**Figure 7:**
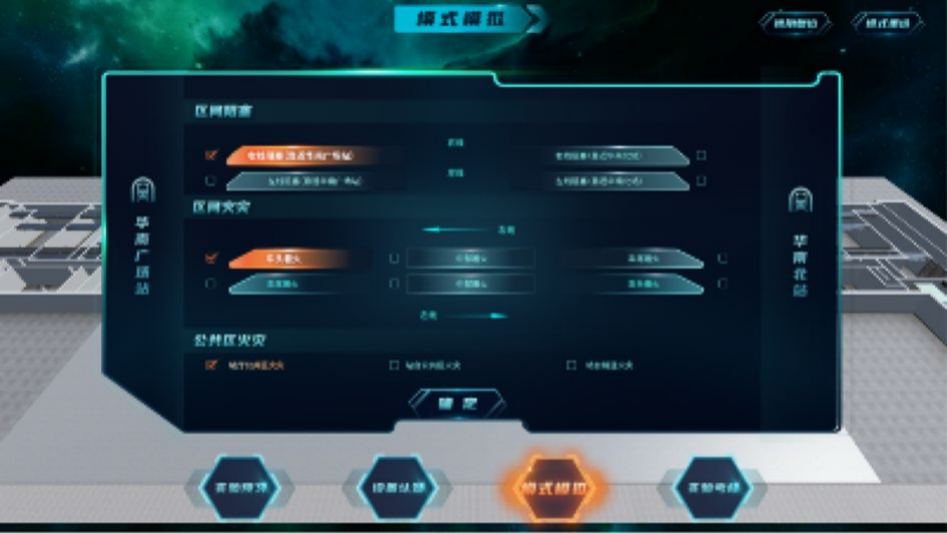
Mode simulation module.

After entering the model simulation module, the system will display three working conditions, section blocking, section tunnel fire, and public area fire. Students need to configure the combined air valve, fan, and other facilities and equipment in the ventilation room according to different scenes to realize the ventilation modes of different scenes, as shown in Fig. 7.

The section blocking conditions include four scenarios: the train is located on the uplink and close to the departure station, the train is located on the uplink and close to the terminal station, the train is located on the downlink and close to the departure station, and the train is located on the downlink and close to the terminal station. The section tunnel fire includes six scenarios: the train is on the uplink, the fire occurs at the head of the train; the train is on the uplink, the fire occurs in the middle of the train; the train is on the uplink, the fire occurs at the tail of the train; the train is on the downlink, the fire occurs at the head of the train; the train is on the downlink, the fire occurs in the middle of the train; and the train is on the downlink, the fire happened at the rear of the train. Public area fire includes three scenarios: station hall fire, platform fire, and platform tunnel fire. The system selects one scene by default for different working conditions. Students can complete one scene experiment under corresponding working conditions or choose other scenes for the experiment.

**Figure 8:**
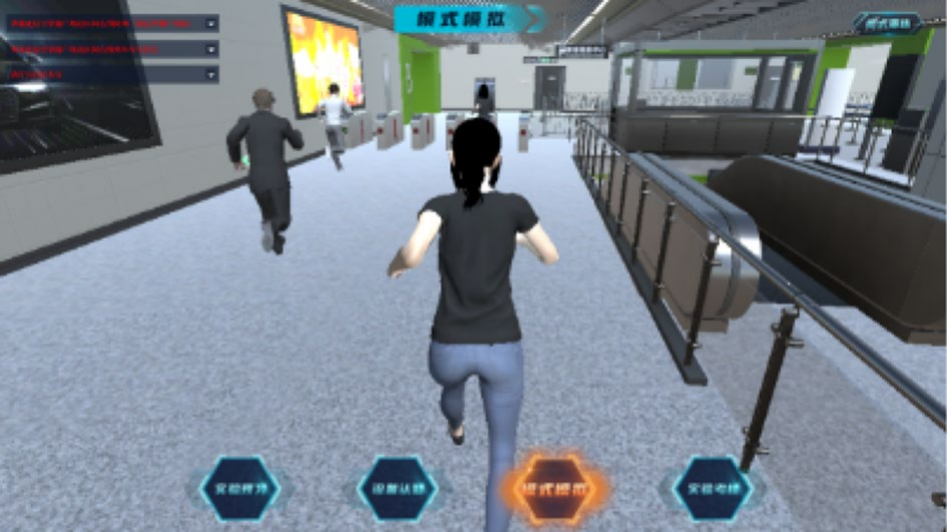
Path roaming.

**Figure 9:**
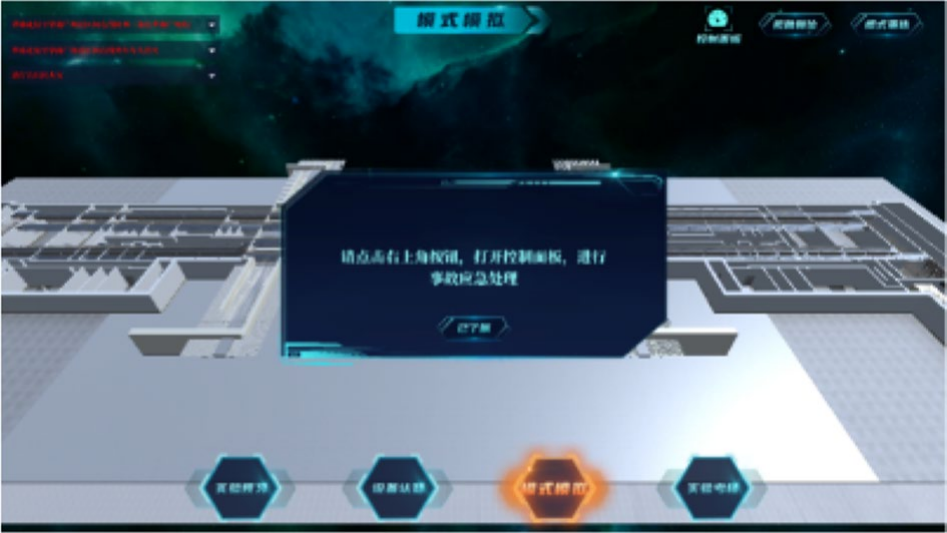
Enter the mode setting page.

Take the fire situation in the public area as an example. After entering the scene, the path roaming will be carried out first. During the roaming, the facilities, equipment, personnel, and smoke in the subway are highly simulated with reference to the real environment, as shown in Fig. 8. After roaming, the system will prompt to open the control panel to complete the mode design, as shown in Fig. 9.

**Figure 10:**
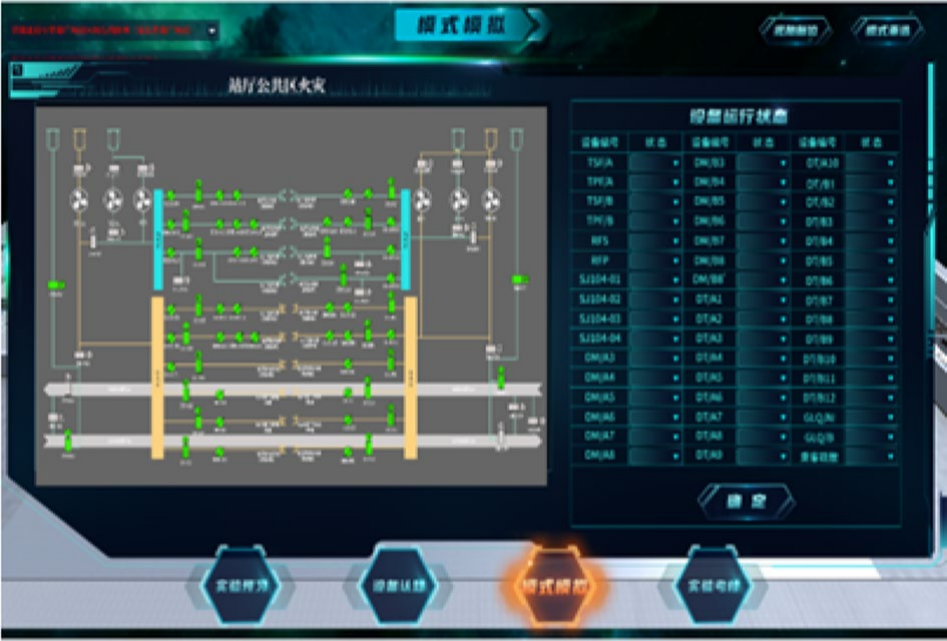
Ventilation control mode design interface.

The combined control of facilities and equipment adopts panel design. The left side of the control interface displays the system diagram of control system facilities and equipment, and the right side displays the combined control mode. The control module can simulate the effect of one button remote control of train dispatching of metro operation company in case of emergency. Students need to control switch of the corresponding equipment to ensure that the control objectives, including four jet fans, up line A and B side of the piston air duct air valve, A and B side downlink circuitous duct air valve, A and B side station blower, exhaust fan, A and B side station A, B side rail against the wind valve, A and B side rail bottom duct air valve, etc., as shown in Fig. 10.

After each working condition is completed, it will enter the next working condition. Click mode reselection in the upper right corner of the interface to reselect the mode. The selected process is the same as the station hall fire in the example public area fire. After completing the correct control mode, the paths of people flow, air flow, and smoke will be displayed.

**Figure 11:**
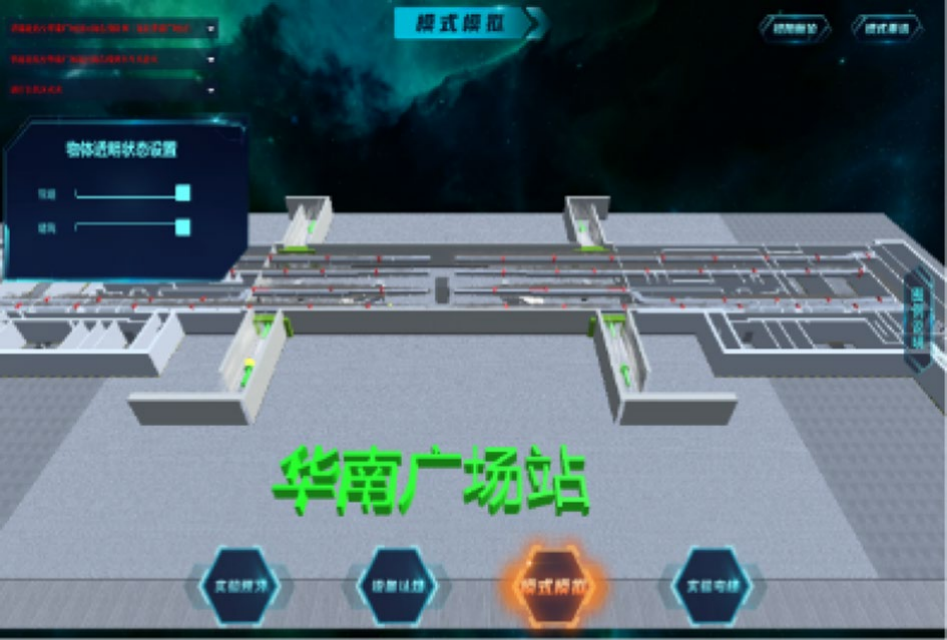
Ventilation system route.

There is only one correct control combination state. The experimenter needs to fully control the state of all equipment and facilities. If the equipment state control is wrong, the system will prompt the wrong control facilities and need to reset the equipment state until all settings are correct, in the case of wrong combination control, the smoke will flow to the unexpected path, which can’t achieve the purpose of rapid smoke exhaust, and thus it is difficult to ensure passengers to escape safely before the hazardous conditions arrive. When the combined control is correct, the system will dynamically display the route of the whole ventilation system, vividly display the flow direction of flue gas and fresh air, and mark the personnel evacuation path, as shown in Fig. 11.

### Platform assessment module

**Figure 12:**
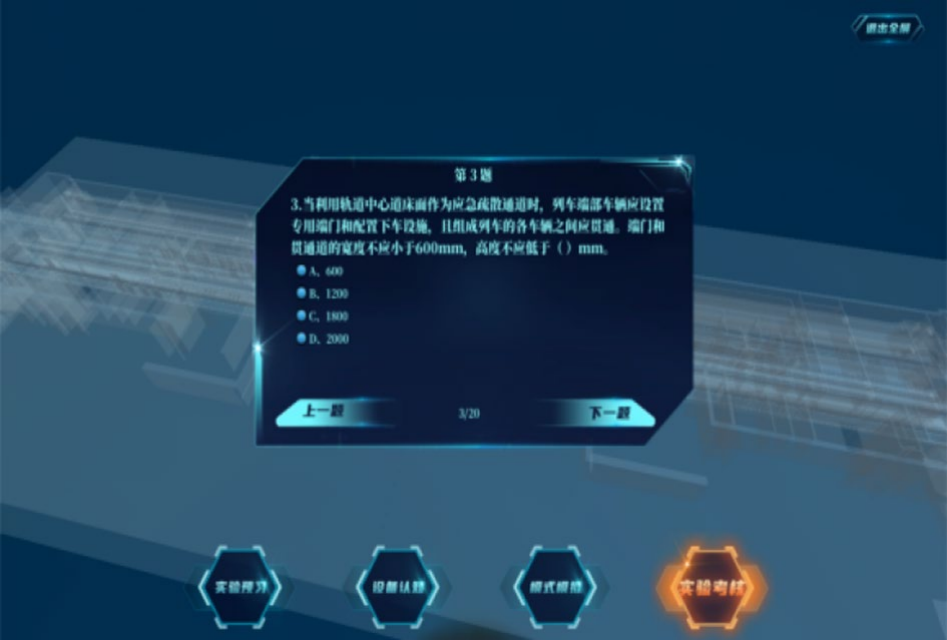
Platform assessment module.

After completing the scene experiments of all working conditions, students will enter the platform assessment module, which requires students to correctly complete the combined control of 47 facilities and equipment states in each mode, correctly determine the escape direction of personnel, and complete 20 basic knowledge topics of ventilation system control within 30 min, as shown in Fig. 12.

## Experimental research on platform teaching effect

### Experimental research object

According to the male female ratio of safety majors in Dalian Jiaotong University, 56 students were randomly divided into an experimental group and a control group, with 23 boys and 5 girls in each group. The experimental group was taught by a virtual simulation experiment teaching platform, and the control group was taught by traditional teaching methods.

Students gave their informed consent to the processing of their data. The study was approved by the Dalian Jiaotong University and was in accordance with the principles of the Declaration of Helsinki.

### Experimental process

**Figure 13:**
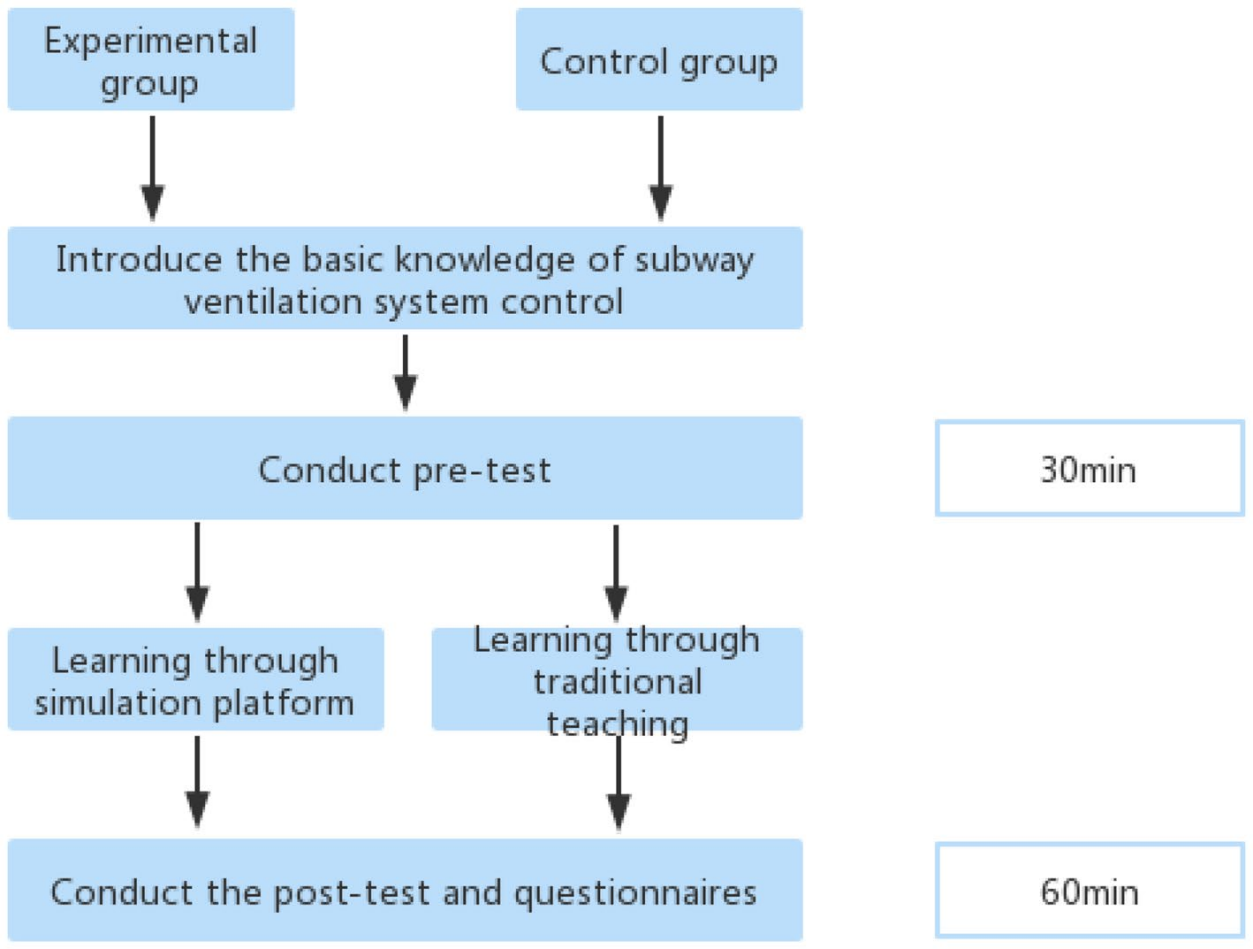
Schematic diagram of experimental design.

1. Students’ basic theory study and pretest before the experiment. The subjects were randomly divided into the experimental group and the control group according to the proportion of male and female students. The basic knowledge of subway ventilation system control was introduced to the two groups of subjects, including the relevant facilities and equipment and their functions, as well as the internal structure of the ventilation system. Conduct pre-test on the experimental subjects to know their understanding of the relevant knowledge of subway ventilation system control. In the pre-test, their cognition of the main equipment in the subway ventilation system and their understanding of the functions of the equipment will be tested. The experimental subjects need to complete these questions within 30 min.
2. Conduct comparative teaching and collect data of practical operation skills. In order to test the operation skills of the experimental objects, after the completion of the experimental pre-test, the objects in the experimental group were taught by a simulation platform, and the objects in the control group were taught by traditional methods. When students use the simulation platform for experiments, the system will record the students’ operation process and the number of errors and automatically judge the scores. The system will provide prompts when students operate, and also record and evaluate the students’ operations. The final score will be determined according to the results of the operation and examination questions. The system will issue an experiment report to those who pass the test, and those who fail will need to conduct the experiment again until they pass the test. The system assessment is used for the operation skill test to measure the students’ cognition and operation ability of the equipment in the ventilation system in case of subway emergencies. The operation skill test can reflect the effect of students’ learning through the simulation experiment teaching platform. Higher scores reflect students’ higher mastering of ventilation system control knowledge.
3. The cognitive load was investigated by questionnaire after teaching. In order to investigate the cognitive load and learning motivation of the subjects, after the completion of the two groups of teaching, the subjects were post tested, and the students’ cognitive load and learning motivation were investigated by questionnaire. The post test will test the students’ cognition of the facilities and equipment in the ventilation system and their ability to control and operate the ventilation system. After that, a questionnaire was used to test the students’ cognitive load and learning motivation. The design process of the experiment is shown in Fig. 13. Experiments and tests are carried out according to the designed teaching and experimental process.

## Student learning effect and test analysis

The research used SPSS tools to conduct an independent sample t-test on two groups of experimental data. Through the research on the test results of students before and after the experiment, this section will analyze the impact of the virtual simulation experiment teaching platform on students’ learning and experimentation.

Cognitive load is the sum of the attention or mental effort a person is using in memory during learning. The study on the cognitive load of the test subjects can reflect the influence of the experimental teaching platform on the experimenter’s learning, the teaching effect of the experiment, and the design effect of the platform content. The cognitive load test consists of 8 items, including "the difficulty of the learning content for the tester" and "whether the learning activity requires a lot of effort to complete the task or goal". The learning motivation test consists of six items, including "whether the experimenter thinks the experiment is interesting and valuable" and "whether the experimenter thinks the experiment is important to everyone". The test of cognitive load and learning motivation refers to the questionnaire designed by Singh^31^.

### Knowledge test analysis

The t-test is conducted on the students’ pre-test results. The Levene test is to evaluate the variance equality of the scores of the two groups. The F value is 1.76 and the *p* value is 0.19, it can be found that there is no significant difference in the average value of the pre-test scores between the experimental group and the control group, as shown in Table 1. The t-test of students’ post-test scores is shown in Table 2. The average score of the experimental group is 93.36 and the average score of the control group is 60.57. The F value is 6.51, the *p* value is 0.01, less than 0.05, indicating that there is a significant difference in the average score of the two groups after the test. Cohen’s D value after the experiment shows that the effect is huge, indicating that the simulation teaching platform has a better effect on students’ operational skills and knowledge learning than traditional teaching.

**Table 1:** T-test of students’ pre-test results.

Group	N	Mean	SD	T	df	*P* Value
Experimental group	28	20.21	4.09	− 0.51	54	0.62
Control group	28	20.71	3.28

**Table 2:** T-test of students’ post-test results.

Group	N	Mean	SD	T	df	*P* Value	Cohen’s d
Experimental group	28	93.36	3.13	29.73	45.75	0	7.95
Control group	28	60.57	4.92				

### Cognitive load analysis

T-test the cognitive load of the two groups of students, as shown in Table 3. Levene test detects the variance difference between the two groups. When the F value is 13.18 and *P* value is 0.00, it indicates that the variance of the two groups is not equal, so the t-test assumes that the variance is not equal. The average cognitive load of the experimental group was 15.43 and the average cognitive load of the control group was 23.79, the *P* value was 0.00, indicating that there was a significant difference in the cognitive load between the two groups, and it was found that the cognitive load of the students in the experimental group was less than that of the students in the control group. Cohen’s D value of the cognitive load is 5.29, indicating that the effect is very strong, and the simulation teaching platform is better than the traditional teaching in reducing students’ cognitive load.

**Table 3:** Student cognitive load t-test.

Group	N	Mean	SD	T	df	*P* Value	Cohen’s D
Experimental group	28	15.43	0.96	− 19.74	38.54	0	5.29
Control group	28	23.79	2.02				

### Analysis of learning motivation

T-test the learning motivation of the two groups of students, as shown in Table 4. Levene test was used to detect the difference in variance between the two groups, F = 7.49, *P* = 0.01, indicating that there was a difference in variance between the two groups. The mean learning motivation of the experimental group was 23.50, and that of the control group was 17.46, with a *P* value of 0.00, indicating that there was a significant difference in learning motivation between the two groups, and the simulation experiment platform had a more positive impact on students’ learning motivation. Cohen’s D value of learning motivation is 2.91, which has a large effect size, indicating that a simulation teaching platform has a more significant positive impact on students’ learning motivation than traditional teaching.

**Table 4:** T-test of students’ learning motivation.

Group	N	Mean	SD	T	df	*P* Value	Cohen’s d
Experimental group	28	23.5	2.53	10.9	43.49	0	2.91
Control group	28	17.46	1.48				

## Conclusion and prospect

Virtual simulation experiment teaching is a new teaching method based on educational environment and teaching reform. Its purpose is to break the limitations of students’ experimental environment and cognition in traditional teaching. Virtual simulation experiment teaching is highly valued and applied in contemporary engineering experiment teaching, which enriches the experiment education, theory teaching, and practice of related engineering majors.

### Conclusion

The purpose of this research is to study the effect of virtual simulation teaching platform on students’ operating skills and cognitive load in engineering laboratory. By analyzing the influence of the virtual simulation experiment teaching platform of ventilation control system on the operating skills and cognitive load of safety engineering students, it is found that the test results of students who adopt the virtual simulation experiment teaching are better than those of traditional teaching methods.

1. The average post test score of the experimental group is 93.36, while the average post test score of the control group is 60.57, which shows that the simulation teaching system can greatly improve the learning effect of students.
2. In the training of operation skills, the students in the experimental group were able to correctly control the status of 47 facilities and equipment under various working conditions and achieved good results, while the students in the control group made some mistakes in the control of facilities and equipment. The application of virtual simulation technology in subway ventilation system control will improve students’ learning of operation skills. It has a more vivid learning environment than traditional teaching methods. It can observe the internal structure and facilities of the ventilation system in 3D, which is helpful for students to understand theoretical knowledge and improve operation skills.
3. Virtual simulation technology has also greatly improved students’ cognitive ability. Through the experimental test, it is found that the cognitive load of the experimental group is 8.36 lower than that of the control group, which is significantly lower than that of the control group. In the virtual simulation experiment, students can simulate the ventilation system control of subway emergencies, observe the ventilation facilities and equipment in an all-round way, immersively view the internal structure of the ventilation system and roam in the station, and control each piece of equipment to complete the ventilation under different working conditions. So that students can approach the real environment to the greatest extent, and provide an experience in which students can better learn and understand the ventilation system, making up for the lack of the traditional classroom.

### Prospect

In Software version 2.0 of the platform, the emergency dispatching and drill knowledge module has been added to expand the emergency drill service function in case of Metro emergencies, so that the system can meet the emergency drill, training, and environmental control personnel learning functions. We firmly believe that the new platform will have better efficiency in the experimental teaching of ventilation control and emergency response ability for subway emergencies.

## Software availability

DTTF software (Registration No.: 2020SR1003565) is protected by copyright. Relevant authorization can be obtained only after the author is registered and authenticated by his real name. We provide proof that the software has been authorized, but we can obtain the right to use the software from the corresponding author upon reasonable request.

## Data Availability

The datasets used and analysed during the current study available from the corresponding author on reasonable request.
